# Peripheral and Spinal Mechanisms of Acupoint Sensitization Phenomenon

**DOI:** 10.1155/2013/742195

**Published:** 2013-09-26

**Authors:** Pei-Jing Rong, Shaoyuan Li, Hui Ben, Liang Li, Ling-Ling Yu, Chang-Xiang Cui, Xia Li, Bing Zhu

**Affiliations:** ^1^Institute of Acupuncture and Moxibustion, China Academy of Chinese Medical Sciences, Beijing 100700, China; ^2^Beijing University of Chinese Medicine, Beijing 100029, China

## Abstract

This study was carried out on adult female Sprague-Dawley rats to observe the position, size, and sensitivity change of inflammatory reactions on body surfaces induced by colorectal import of inflammatory irritant mustard oil. Colorectal distension (CRD) was adopted as a visceral noxious stimulus to record the activities of spinal dorsal horn wide-dynamic range (WDR) neurons activities at spinal segments L1–L3. The study also observed the activations of WDR neurons by electro-acupuncture (EA) on acupoints of Zusanli-Shangjuxu before and after different intensities of CRD stimulation and the dose-response relationship between stimulus and response. The results show that in the case of visceral inflammation, the number of exudation points of neurogenic reaction on body surfaces increased along with the severity of visceral inflammation (Li et al. 2006). The area of peripheral receptive fields of WDR neurons also enlarged along with the intensity of visceral inflammatory response. The activation effect of EA on WDR neurons was positively correlated with the severity of visceral inflammation. Therefore, we concluded that the function of acupoints can be sensitized by visceral noxious stimuli. When the function of internal organs was damaged, the number of reaction points on body surfaces, the size of acupoints' receptive fields, and the sensitivity of acupoints changed accordingly.

## 1. Introduction

Acupoints are special locations on body surfaces where the Qi of viscera is transfused. It is the key step underlying the interaction between meridians and viscera. Visceral diseases can induce mechanical hyperalgesia on the corresponding acupoints on body surfaces which is manifested as pain when pressed. The size and function of acupoints change accordingly with the change of the visceral functions. The diagnostic and therapeutic effects of acupoints on splanchnic diseases enhance in pathological conditions. This phenomenon is called “acupoint sensitization”.

A previous study [1] demonstrated that when splanchnic disease occurred, acupoints on body surface turned from the silent mode in physiological condition to the activated or sensitized mode in pathological condition. When acupoints are activated or sensitized, the regulatory or therapeutic effects of acupuncture on corresponding viscera change in both quality and quantity. At the present, the mechanism behind acupoints sensitization phenomenon is largely unknown. Adopting colorectal import of inflammatory irritant mustard oil and the colorectal distension (CRD) as the method of visceral noxious stimulation, this study observed the body surface inflammatory reactions and activities of wide-dynamic range (WDR) neurons, explored the dose-response relationship of acupoint sensitization, and investigated the regulatory effect of acupuncture in acupoint sensitization and related spinal cord mechanism.

## 2. Method

### 2.1. Experiment of Inflammatory Exudation Points

#### 2.1.1. Experimental Animals

20 clean level healthy male Sprague-Dawley rats, weighing 250–300 g, were obtained from the Laboratory Animal Center of the Military Academy of Medical Sciences.

#### 2.1.2. Induction of Inflammatory Condition

This study used the method of colorectal import-inflammatory irritant mustard oil. A tube was inserted into the rat's colon and rectum through anus at the depth of 2 cm to 3 cm, and different doses of 2.5% mustard oil (mustard oil; Sigma-Aldrich, St. Louis, MO) were inserted when needed.

#### 2.1.3. Exudation Points of Neurogenic Inflammation on Body Surfaces

Evens blue (EB) was injected (5 mg per 100 g body weight of rat) into the rats' tail vein to mark the exudation points of neurogenic reaction on body surfaces. The concentration was 50 mg/mL.

### 2.2. Experiment on Spinal Cord

#### 2.2.1. Animals and Surgical Procedures

43 clean level healthy male Sprague-Dawley rats, weighing 250–300 g, were obtained from the Laboratory Animal Center of Military Academy of Medical Sciences. Rats were fast for 12 hours before experiment but water was not deprived.

Rats were anesthetized with an intraperitoneal injection of 10% urethane (1.0~1.2 g/kg) and then were placed in supine position on the operating table for tracheal intubation. One hundred *μ*g atropine was administered through intraperitoneal injection to reduce secretions from the trachea. Rats were placed in the stereotaxic apparatus after operation, injected intraperitoneally of 2% gallamine triethiodide (2 mL/rat), and connected to the breathing machine (Parameters: tidal volume: 4 mL/100 g; respiratory rate: 60 breaths per minute; respiratory quotient: 1 : 1). The body temperature of experimental rats was maintained at 37°C by electric blanket during the operation and experiment. Rats were also anesthetized during colorectal infusion of mustard oil.

The skin around the waist of the rats was cut along the midline of the back to expose and fix the spinal segments L1–L3. The coordinate of WDR neuron is 0.5–1.5 mm beside the midline of the back of spinal cord and 500–1500 *μ*m beneath the surface of spinal cord. A microelectrode was used to record the neuron discharge activity, and the electrical signals were collected and processed by microelectrode amplifier and PowerLab electrophysiological recording system.

#### 2.2.2. Identification of WDR Neurons

Neurons located in the spinal dorsal horn that respond to given noxious stimulations (e.g., clamping skin, CRD and so on) and nonnoxious stimulations (e.g., brushing, touching skin, and so on) with electric discharge are called WDR neurons. Only WDR neurons were conducted as objects of this study.

Seventy-seven WDR neurons were recorded at the spinal segments L_1~3_ of the 43 experimental SD rats in this study.

#### 2.2.3. Visceral Noxious Stimulation

Colorectal distension (CRD) was adopted in the experiment. A 4 to 6 cm long balloon made from a disposable condom tip was tied to on a 4 mm diameter hose. The other end of the hose was connected to the sphygmomanometer and pressure transducer through the T-type channel. In the experiment, the balloon was inserted into the rat's straight colon through anus to the depth of about 4 cm to avoid direct stimulation to anus and bowel wall. Before the balloon was placed into the colon, 3–5 drops of tepid paraffin oil were smeared on the balloon' surface. The distance from balloon end to anal was about 0.5 cm. A pressure of 20–80 mmHg was applied through the sphygmomanometer with the duration of about 20 s. Pressures of more than 40 mmHg were regarded as visceral nociceptive stimulus [2, 3]. The interval between two CRD stimulations was kept more than 10 min to avoid possible sensitization of colon caused by overstimulation.

#### 2.2.4. Electroacupuncture

Electroacupuncture was applied at the acupoints of “ZUSANLI-SHANGJUXU” at the recorded homolateral discharging neurons with the frequency of 20 Hz for 30 seconds. The intensity was 1.5 times of the intensity of the A*δ* fiber threshold stimulus (about 1.82 ± 0.64 mA [4]).

### 2.3. Experiment Process

#### 2.3.1. Observation of Exudation Points of Neurogenic Reaction on Body Surfaces

Twenty rats were used in the experiment. Different doses of mustard oil (20, 50, 100, 150, 200 *μ*L) were imported into the rat's colon. Two-three hours after importing, evens blue (EB) was injected into the rats' tail vein as the marking pigment [5]. The distribution and size of exudation points (blue points) of neurogenic reaction on body surfaces were observed and recorded after different doses of mustard oil were imported.

#### 2.3.2. Observation of Colorectal Inflammatory Reaction and the Size of Receptive Fields

11 rats were used in the experiment. At least 4 points which could increase the discharge of WDR neurons were measured and identified by the acupuncture needle along the horizontal and vertical axes at the acupoints of ZUSANLI-SHANGJUXU, and the size of peripheral receptive field was marked. Fifty *μ*L and 200 *μ*L of mustard oil were imported into the colon of rats successively to induce colorectal inflammatory reaction. Then, acupuncture was used again as the probe to detect and record the discharging reactions of WDR neurons and the size of peripheral receptive fields. The purpose was to observe the changes of WDR neurons at peripheral receptive fields before and after the colorectal inflammatory reaction.

#### 2.3.3. Effect of Different CRD Stimulations on the Discharging Reactions of WDR Neurons

Thirty-two rats were used in the experiment. The discharging reactions of WDR neurons at ipsilateral peripheral receptive fields of “ZUSANLI SHANGJU XU” acupoints were recorded. Different levels of CRD stimulations (nonnoxious stimulations: 20 mmHg; light noxious stimulations: 40 mmHg; moderate noxious stimulations: 60 mmHg and strong noxious stimulations: 80–100 mmHg) were applied to rats to observe the discharging reactions of WDR neurons after stimulations as well as the dose-effect relationship between CRD stimulation and WDR neuron reaction.

#### 2.3.4. Observation of the Effect of Acupuncture on Discharging Reactions of WDR Neurons

Electroacupuncture was administered for 30 seconds before and after the application of different intensities of CRD stimulation. We recorded the discharging reactions of WDR neurons and observed the effect of acupuncture on WDR neuronal discharging reactions before and after CRD stimulations.

### 2.4. Histological Location

**Figure 1:**
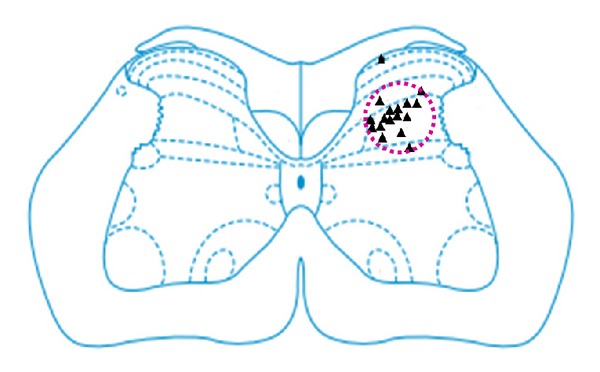
The locations of identified WDR neurons in the spinal cord. ▲ indicates the location of a neuron.

After the single cell recording, pontamine sky blue was imported from a glass microelectrode into the DCN neuron group to mark the position of electrode's recording by passing 20 *μ*A of negative direct current through the microelectrode for 20–30 min by the microelectrode amplifier. Then, the rats were executed and perfused through the heart with 4% of paraformaldehyde. The rats' lumbar spinal cord was removed and fixed in the paraformaldehyde. Two days later, the lumbar spinal was made into frozen sections for H and E staining. Locations that were not recorded in the DCN neuron group were weeded out. As shown in Figure 1, most WDR neurons marked in this experiment were located in Rexed layers IV and V, and a few were in Rexed layers I and VI.

### 2.5. Data Collection and Analysis

Software such as Powerlab data acquisition system, Chart 5.0, and SPSS13.0 were used for data collection and analysis. The volume of neuronal discharge per second and the activation/inhibition rate were calculated. The mean and standard deviation before and after the electroacupuncture intervention were calculated as the descriptive statistics and represented by $\overset{-}{X}\pm \text{SE}$. The activation/inhibition rate was represented by $\overset{-}{X}\pm \text{SE}$%. The independent *t* test was used for the comparison between groups. *P* < 0.05 was considered as statistically significant.

## 3. Results

### 3.1. Dose-Effect Relationship between Different Doses of Mustard Oil Imported into the Colon and the Exudation of Neurogenic Reaction on Body Surfaces

Two hours after the injection of mustard oil, colonic mucosa hyperemia and edema were viewed clearly under low power lens (×40). Sporadic and abundant polymorphonuclear leukocytes, tissue damage, and bruises were viewed on the mucous membrane under high power lens (×400), but the same expression was not viewed in the saline injection group.

**Figure 2:**
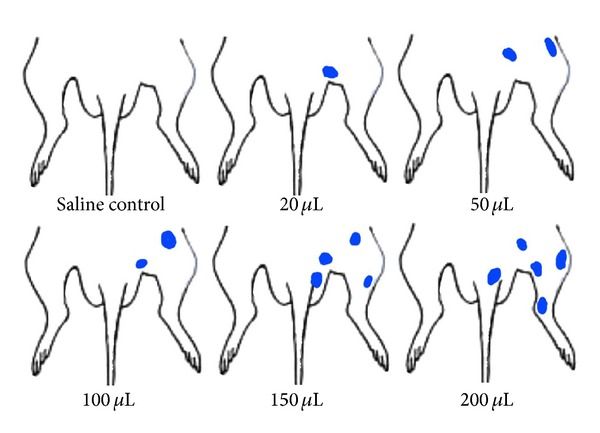
The distribution of EB exudation points on body surfaces after injection of mustard oil into the colon.

After the injection of different doses of mustard oil into the colon, the number of EB exudation points increased along with the dose of mustard oil. Generally, 0–2 EB exudation points were observed with the dose of 20 *μ*L, 1–3 points with the dose of 50 *μ*L, 2-3 points with the dose of 100 *μ*L, and 2–5 points with the dose of 150 *μ*L. When the dose was increased to 200 *μ*L, EB exudation points increased to 3 ~ 7. It indicated that the number of exudation points is in proportion to the inflammatory reaction level of organs (Figure 2). Exudation points were largely located in the lower extremities of the body, crotch, and pars basilar is of tail. Whereas, in the rats group injected with equal doses of saline, no regular EB exudation points was observed.

### 3.2. Changes of Peripheral Receptive Field of WDR Neuron in Spinal Dorsal Horn in the Colorectal Inflammatory Reaction

**Figure 3:**
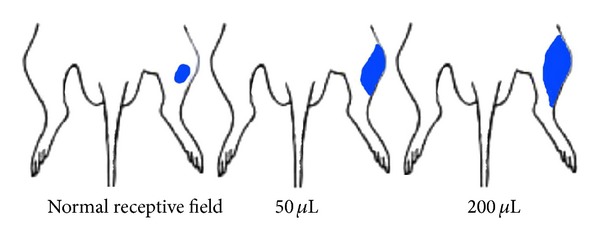
The peripheral receptive field of WDR neurons in the spinal cord after the injection of mustard oil into the colon.

We recorded 11 WDR neuronal discharge activities at L_1–3_. Normally, the peripheral receptive field of this type of neurons is relatively small, with an average size of 0.61 ± 0.17 cm^2^. After the injection of 50 *μ*L mustard oil, the average size of peripheral receptive field increased to 0.85 ± 0.43 cm^2^. When the dose of mustard oil increased to 200 *μ*L, the average size of peripheral receptive field increased to 1.13 ± 0.87 cm^2^. It showed that the receptive field transformed from the relatively small size under the physiological condition to the relatively big size under the pathological condition (Figure 3).

### 3.3. The Activating Effect of CRD Stimulation on WDR Neurons

**Figure 4:**
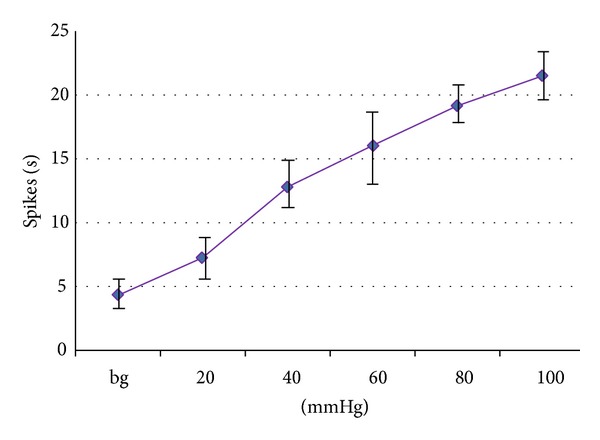
Activating effect of different intensities of CRD stimulation on WDR neurons.

We recorded 8 WDR neurons and observed their discharge reactions induced by CRD stimulation at 20, 40, 60, 80, and 100 mmHg. The result is that the background discharge of WDR neurons increased gradually from 4.32 ± 1.13 spikes/s to 21.42 ± 2.68 spikes/s after CRD stimulation at 100 mmHg. The increasing rate was 495.83 ± 43.53% of background discharge (*P* < 0.05 ~ 0.001). It demonstrated that visceral noxious stimulation notably activated WDR neurons and increased the number of neuronal discharge per unit time (Figure 4).

### 3.4. Effect of Acupuncture on the Discharge Activities of WDR Neurons before and after CRD Stimulation

**Figure 5:**
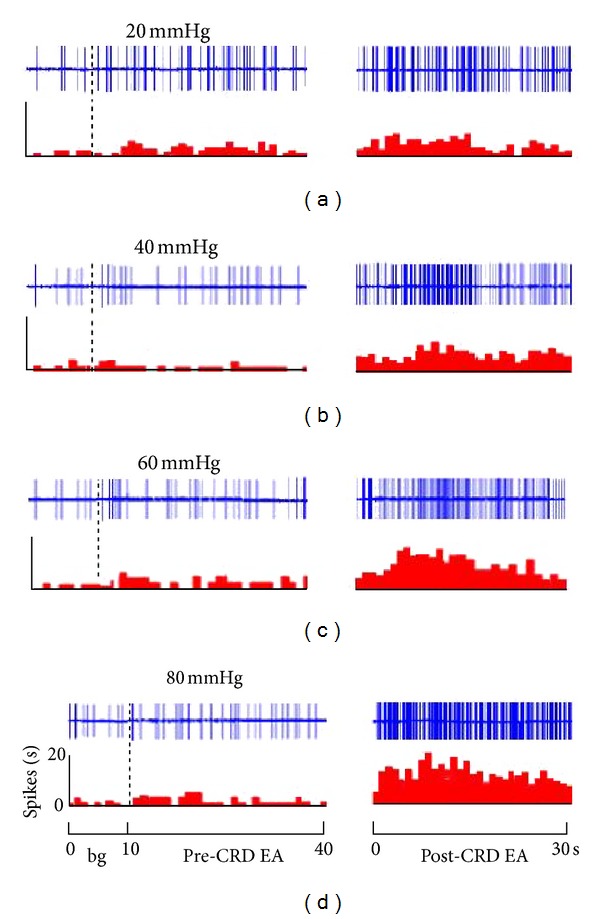
The responses of WDR neurons to EA before and after CRD. Note: upper rows showing original unit discharges and lower rows showing histograms.

EA was applied to rats before and after different intensities of CRD stimulations. Results showed that as the intensity of CRD stimulation increased, its activating effect on WDR neurons in spinal dorsal horn also increased remarkably. No obvious change was observed in the discharge activities of WDR neurons if EA was given before the CRD stimulation. If EA was given after the CRD stimulation, its activating effect on WDR neurons was notably enhanced (Figure 5).

The activating reaction induced by EA was observed in 15 WDR neurons at 20 mmHg CRD (Figure 5). Before CRD, WDR neurons had no activating reaction to EA stimulation compared with background activity (*P* > 0.05). After CRD, EA had significant activating effect on WDR neurons. There was a significant difference before and after CRD (*P* < 0.05).

The activating reactions induced by the same intensity of electroacupuncture were observed in 14 WDR neurons at 40 mmHg CRD. The result showed that the neuronal discharge increased 43.38 ± 3.67% after CRD compare to that before CRD. There was a very significant difference (*P* < 0.001).

Sixty mmHg and 80 mmHg CRD were applied to WDR neuron No. 13 and No. 14, respectively, to observe the activating effect of EA. The result showed that after high intensity noxious stimulation, discharging activities induced by EA increased 71.61 ± 8.82%, and 94.32 ± 10.41% respectively, and the difference was significant (*P* < 0.001).

**Figure 6:**
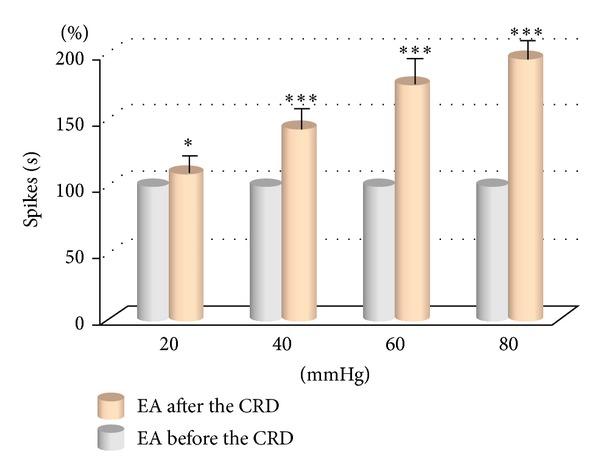
The response of WDR neurons to EA at different CRD. *(*P* < 0.01); ***(*P* < 0.001).

It is clear that at the range of 20 to 80 mmHg, the activating effect of EA of the same intensity on WDR neurons increased as the CRD intensity increased. CRD intensity and the EA activating effect presented a linear quantity-effect relationship (Figure 6).

## 4. Discussion

The connection between acupoints and viscera is bidirectional and reciprocal. The gut-associated acupoints on body surfaces can treat corresponding visceral diseases. It is also a mirror that specifically reflects the visceral function and the QI-XUE change of the human body [6]. In pathologic state, the connection between internal organs and acupoints is closer than in normal condition [7]. When diseases occur in internal organs or deep tissue, visceral nociceptive afferent can facilitate the somatic afferent. hyperalgesia and skin sensitization occur at corresponding acupoints or positions on body surfaces, which reflects in the increase and relative concentration of the tender points or sensitive points on the skin [8].

Recently, some researchers proposed the idea that acupoints are dynamic, that is, the size and function of acupoints are not in the static state; the functioning of acupoints is an active and dynamic process. The function and size of acupoints change with the state of the body, especially the function of their corresponding internal organs [6, 9]. In other words, as the visceral function changes, the functional activity of acupoints switches from the silent mode in physiological condition to the activated or sensitized mode in pathological condition [10, 11].

Our experiment showed that with visceral inflammatory reaction caused by colorectal injection of inflammatory irritant mustard oil, the number of EB exudation points and the average size of receptive field of WDR neurons on body surfaces increased as the visceral inflammatory reaction intensified. The number of exudation points and the size of receptive field were in proportion to the degree of visceral inflammatory reaction. Some researchers presented that C fibers might mediate this neurogenic exudation reaction [12]. Others thought exogenous noxious small organic molecules, such as mustard oil, could activate ion channel of pain TRPA1 [13, 14]. When tissue was damaged, some endogenous compounds might also activate TRPA1 [15] and lead to the increase of permeability and hyperalgesia.

Experiments confirmed that 20–100 mmHg CRD stimulations obviously increased (activated or sensitized) reactions of WDR neurons in the spinal cord L_1~3_ induced by noxious and non-noxious stimulations to the receptive field. When EA was applied at the receptive field after CRD stimulation, the discharging activity of WDR neurons notably increased compared to that before the CRD stimulation and the receptive field also expanded. Meanwhile, the discharging activity of WDR neurons increased as the CRD intensified. The two factors presented an obvious linear quantity-effect relationship. It indicated that visceral noxious stimulation caused the sensitization of corresponding acupoints (or positions) on body surfaces and further enhanced the effect of acupuncture.

It is generally believed that when visceral lesions occur, the abnormal phenomenon of hyperpathia on acupoints is the result of the facilitation or sensitization of the function of spinal cord and/or the center up spinal cord. Afferent impulse in internal organs or deep tissue can sensitize wide dynamic range (WDR) neurons and facilitate stronger reactions of the neurons to the afferent nerve on body surfaces [16, 17]. Repeated stimulation to the peripheric receptive fields of neurons in the spinal cord dorsal horn can also lead to the expansion of the receptive field [18]. It suggests that the mechanism behind the change of perigheric receptive field of sensory neurons may relate to the change of acupoint size.

## 5. Conclusion

Visceral noxious stimulation can increase the number of EB exudation points, reduce the reaction threshold of WDR neurons in the spinal cord dorsal horn, increase background discharge levels, enlarge the receptive fields of neurons, and sensitize the function of acupoints. It demonstrated that the pathological change of internal organs' functional activity lead to the sensitization of spinal center and further to the changes of the size and function of acupoints on body surfaces. The spinal cord takes part in the dynamic process of acupoint sensitization. A linear quantity-effect relationship can be found between the noxious stimulation and the body response.
